# Estimating the COVID-19 prevalence from wastewater

**DOI:** 10.1038/s41598-024-64864-1

**Published:** 2024-06-22

**Authors:** Jan Mohring, Neele Leithäuser, Jarosław Wlazło, Marvin Schulte, Maximilian Pilz, Johanna Münch, Karl-Heinz Küfer

**Affiliations:** Fraunhofer Institute for Industrial Mathematics, 67663 Kaiserslautern, Germany

**Keywords:** Covid-19, Cohort study, Wastewater-based epidemiology, Mathematical modelling, Forecast, Computational biology and bioinformatics, Biomarkers, Diseases, Health care, Mathematics and computing

## Abstract

Wastewater based epidemiology has become a widely used tool for monitoring trends of concentrations of different pathogens, most notably and widespread of SARS-CoV-2. Therefore, in 2022, also in Rhineland–Palatinate, the Ministry of Science and Health has included 16 wastewater treatment sites in a surveillance program providing biweekly samples. However, the mere viral load data is subject to strong fluctuations and has limited value for political deciders on its own. Therefore, the state of Rhineland–Palatinate has commissioned the University Medical Center at Johannes Gutenberg University Mainz to conduct a representative cohort study called SentiSurv, in which an increasing number of up to 12,000 participants have been using sensitive antigen self-tests once or twice a week to test themselves for SARS-CoV-2 and report their status. This puts the state of Rhineland–Palatinate in the fortunate position of having time series of both, the viral load in wastewater and the prevalence of SARS-CoV-2 in the population. Our main contribution is a calibration study based on the data from 2023-01-08 until 2023-10-01 where we identified a scaling factor ($0.208 \pm 0.031$) and a delay ($5.07 \pm 2.30$ days) between the virus load in wastewater, normalized by the pepper mild mottle virus (PMMoV), and the prevalence recorded in the SentiSurv study. The relation is established by fitting an epidemiological model to both time series. We show how that can be used to estimate the prevalence when the cohort data is no longer available and how to use it as a forecasting instrument several weeks ahead of time. We show that the calibration and forecasting quality and the resulting factors depend strongly on how wastewater samples are normalized.

## Introduction

In late summer 2022, it became politically clear that preventive mass testing for SARS-CoV-2 was coming to an end. Neither the high costs nor the intrusion into the private lives of citizens could be justified by the now low threat. An end to mass testing also meant that politicians would lose any overview of the infection situation, even if the threat were to increase again. So a cost-effective and socially less invasive alternative was sought. In Rhineland–Palatinate, the Ministry of Science and Health therefore consulted the University Medical Center Mainz and the Fraunhofer Institute for Industrial Mathematics. Both institutions had already successfully navigated the pandemic before. They came up with a surveillance strategy consisting of three components: a representative cohort study with 14,000 participants, wastewater monitoring at 16 sewage treatment plants evenly distributed across Rhineland–Palatinate, and an epidemiological model for fitting and forecasting.

A major goal was to calibrate the cost-effective and long-term wastewater monitoring over a period of several months against the expensive but informative cohort study in order to find a conversion factor between the quantities *viral load* and *prevalence*. The prevalence can help politicians to estimate the load on hospitals or the loss of working hours. A similar feeling for the consequences is missing for wastewater data. Therefore, a conversion factor is needed translating viral load into prevalence. To get this factor we proceed as follows. First, the parameters of a simple epidemiological model are adjusted so that the simulated curves for viral load and prevalence are as close as possible to those measured during the calibration period. This gives the desired conversion factor between the two curves, which are scaled and shifted copies of each other by construction of the model. Later, only measurements of the viral load will be available. In this situation the epidemiological model is only adapted to the wastewater data. The result is a smooth simulated curve interpolating the measured viral load. If this curve is now multiplied by the previously found conversion factor, we can finally reconstruct the unknown prevalence.

A purely data-based approach that fits a non-parametric regression model to predict the cohort study prevalence from wastewater data did not prove to suffice to explain the inherent connection between these data sources^1^. However, when integrated with the epidemiological model, the forecasting strength becomes apparent.

Wastewater-based epidemiology (WBE) has become more and more popular in the last decade as a cost-efficient means of detecting all kinds of markers such as pesticides, drugs or diet markers. Biological markers only played a subordinate role then^2^. The early research provides evidence that the elements can indeed be found in the wastewater samples, but were usually used as a binary indicator of whether or not a pathogen is present^3,4^. Later, also quantitative analyses were made and detected virus loads were compared to observed clinical cases^5^, however, the clinical data was typically sparse. With the beginning of the global SARS-CoV-2 pandemics, many more countries and regions have implemented a wastewater surveillance system in order to scan for the existence or the amount of virus in the local sewage system^6–8^ and visualized the data to the public^9^. Since the data of reported cases has become more granular and available, many research groups have compared the detected viral loads with the regional case numbers statistically^10^. Modellers have also exploited the detected viral load in their SEIR-based model in order to calibrate it^11^. Statistical regressions have been performed in order to compute reported cases based on the measured viral load^12^. The challenge that arises is, that both the wastewater data and the number of reported cases are highly error-prone. The measured viral load varies due to numerous sources of uncertainty^13,14^.The number of tests has also always been influenced and thus distorted by accessibility and political and social pressure to get tested^12,15–17^. However, little effort has been made to calibrate the wastewater data to representative cohorts. There are previous known studies, where viral load was sampled locally e.g. at university campus sewersheds and the data was compared to the number of known infected cases in the population because the students were tested regularly as part of a screening programme^18,19^. These valuable comparisons show the high correlation of wastewater viral loads to the real number of cases. There is also one known study in a larger population where a representative sampling of households was used for a calibration of wastewater data^20^.

Our contribution is a comparison and validation of wastewater viral load data and case numbers of a representative cohort based on a new SIR-like model that allows us also to forecast the case numbers beyond the available wastewater data. We are also presenting the first such study for Omicron variant, since the formerly mentioned studies took place in 2021, when Alpha, Beta and Gamma variants were dominant. The setting is also very different. While McMahan et al.^18^ were able to match the wastewater data of an isolated campus with the known (PCR confirmed) infected individuals, we only have self-reported tests of a representative sample of some of the cities. However, this sample is on average much larger than the representative samples that were taken in the Oregon household study in Layton et al.^20^. The wastewater samples in both of these comparable studies used a time proportionate sampling method of 24-h composite samples. None of these used a reference virus for normalization, but the flow normalization (denoted as *copy rate*)^18^ and the concentration^20^, respectively. Only the former has two time series of measurements of prevalence and viral load for the calibration available and also uses them to calibrate a SEIR model. Both studies highlight a fact, that matches with our findings. While the correlation between genecopies in the wastewater and reported cases is often not so strong^21^, the correlation to a representative or even exact number of cases is indeed very strong. While our results have only been tested on SARS-CoV-2 data, WBE has also been successfully applied to other pathogens like Influenza^22–24^, RSV^22–24^ or other common respiratory viruses^24^. Although the detection quality and the stability of different genes varies, our approach of calibrating the measurements with an epidemiological model and a sentinel cohort can in general be transferred to other pathogens as well.

**Figure 1:**
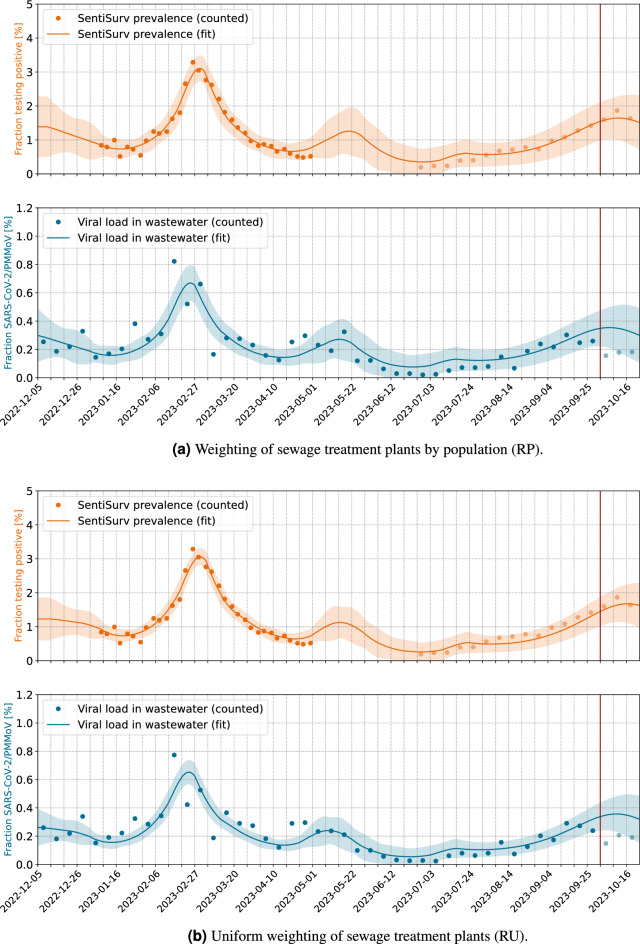
Reconstruction of prevalence from viral load in wastewater. Normalization by PMMoV. The vertical red line marks the date of model fitting. Pale dots indicate measurements which do *not* enter the fit, but are used for validation. These are viral loads measured after the fit and all prevalence data of phase II of the SentiSurv study.

## Results

While the relevance of WBE is widely accepted, it is still a major concern that data can not be compared between sites and no prevalence can be derived^25^. Most dashboards therefore focus on the visualization of trends only^26^ and/or state appropriate warning messages on their website^27,28^. Here we show that more precise results are well possible if representative comparative data (rather than only officially reported cases) are initially collected.

### Prevalence can be reconstructed from wastewater using a model calibrated with a cohort study

Since 2023, the following favorable conditions exist in Rhineland–Palatinate: 16 wastewater treatment plants provide two samples per week. They can be normalized with respect to the reference virus PMMoV (Pepper mild mottle virus), which is analyzed simultaneously. This is a plant RNA virus that is known to be a good indicator for the ratio of human originated wastewater^22,29,30^. Alternatively, the viral load can be normalized by the daily volume flow through the sewage plant which, however, proved to be less reliable. Moreover, there are two options to aggregate the normalized viral loads from the different sewage plants. Firstly, one can weight them equally (“uniformly weighted”). Secondly, one can weight them according to the population connected to the sewage plants (“population-weighted”). The weekly averages of the aggregated, normalized values over all plants, still noisy, are interpolated by an epidemiological model. This model has been calibrated from January to April with prevalence data from an extensive cohort study.

Using the PMMoV-normalization, it is possible to reconstruct the prevalence in Rhineland–Palatinate quite accurately for the validation period (July to September) from the wastewater data, by subsequently adapting the simulation to these data only. This observation holds for both weighting approaches of the different sewage plants.

**Table 1:** Overview over the four different scenarios for wastewater data processing.

Scenario ID	Aggregation method	Normalization method
RP	Population-weighted	Reference virus (PMMoV)
RU	Uniformly weighted	Reference virus (PMMoV)
VP	Population-weighted	Volume flow
VU	Uniformly weighted	Volume flow

**Figure 2:**
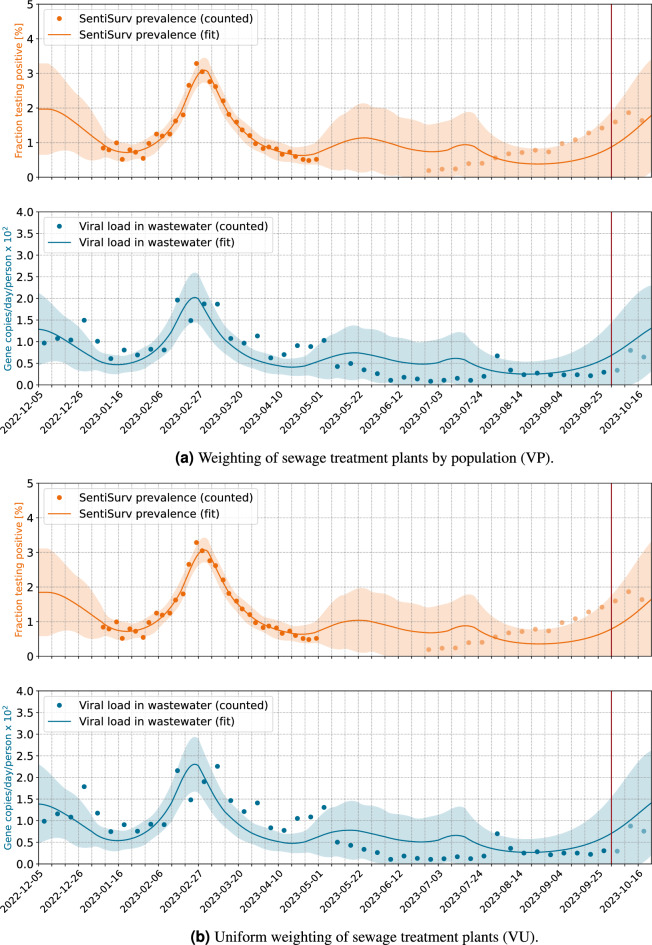
Reconstruction of prevalence from viral load in wastewater. Normalization by volume flow. The vertical red line marks the date of model fitting. Pale dots indicate measurements which do *not* enter the fit, but are used for validation. These are viral loads measured after the fit and all prevalence data of phase II of the SentiSurv study.

The results are illustrated in Figs. 1 and 2 with the scenarios being explained in Table 1. The ratio of gene copies of SARS-CoV-2 to gene copies of PMMoV is plotted as blue dots. Each dot represents the averages over two samples per week and over all 16 sewage treatment plants. The plants are weighted by population in Fig. 1a and uniformly in Fig. 1b. There are only minor differences. The orange dots mark the prevalence measured in the SentiSurv study for the five biggest cities of Rhineland–Palatinate.

In the background, an epidemiological model is adapted simultaneously to both, the wastewater data and to the SentiSurv data of phase I (bold orange). In the following, we will also use the terms adjust, calibrate or fit as synonyms for adapt. One state variable in our model is the proportion *i* of infectious persons. This number cannot be measured directly but we assume that it is represented by the SentiSurv and wastewater values in different manners. Specifically, we assume that the true prevalence *s* is just this proportion *i*, shifted by a few days (delay $\Delta$), and that the true viral load *w* is just a scaled version of that proportion (with scaling factor $\gamma$). Here, *s* stands for SentiSurv and *w* stands for wastewater.

1 $$\begin{aligned} i&:= \frac{\text {Infectious people in the entire population}}{\text {Size of population}} \end{aligned}$$

2 $$\begin{aligned} s&:= \frac{\text {People in the representative cohort testing positive}}{\text {Size of representative cohort}} \end{aligned}$$

3 $$\begin{aligned} w&:= \frac{\text {Mean number of gene copies of sections }N1\text { and }N2\text { of SARS-CoV-in the wastewater sample}}{\text {Gene copies of PMMoV in the wastewater sample}} \end{aligned}$$

4 $$\begin{aligned} s(t)&= i(t-\Delta ) \end{aligned}$$

5 $$\begin{aligned} w(t)&= \gamma \; i(t)\; . \end{aligned}$$

The fitted model provides predictions of prevalence and viral load, which are plotted as solid lines.

The really interesting parts of the figures refer to the prevalence during phase II of the SentiSurv study (light orange dots). Although these measurements have *not* been used for fitting the model, they are well interpolated within the computed 2$\sigma$ error envelope (light orange area, 95% CI). Obviously, it is enough to fit the smooth spreading dynamics to the noisy wastewater data, once the scaling factor between viral load and prevalence is found.

Fitted values of the scaling factor $\gamma$ and the time delay $\Delta$ in Eqs. 4 and 5 are shown in Table 2. In particular, the scaling factor $\gamma$ remains the same within the estimation error, if adjusted only to phase I of the SentiSurv study (winter, no plot) or to both phases (winter and summer, Fig. 1). This means that the scaling factor has not changed over the investigated period from January to September 2023 and seems to be an invariant of the variants of SARS-CoV-2 present at that time.

To be more precise, if (1) the viral load is measured as quotient of the mean number of gene copies of the sequences *N*1 and *N*2 of SARS-CoV-2 and of the number of gene copies of PMMoV, (2) the prevalence is measured as the fraction of people testing positive with an antigen test as sensitive as Verino®Pro and (3) the virus variants behave like those predominant between January and September 2023, then the scaling factor between the two time series is a constant.

The delay between the viral load in wastewater and the prevalence among tested people is probably less than a week. This means that waste water surveillance does *not* provide a significant advantage over rapid tests in early detection of new waves. This finding is in line with the comparison within a controlled setting^18^ and with earlier analysis that the lead-time towards reported cases is heavily influenced by the testing behaviour of the control group^31^.

**Table 2:** Parameters relating viral load and prevalence.

Scenario ID	Fitting period	Scaling$^{\textrm{c}}$ $\gamma$	Delay$^{\textrm{c}}$ $\Delta$ (d)
RP	Phase I$^{\textrm{a}}$	0.220 ± 0.038	5.05 ± 2.34
RP	Phase I &II$^{\textrm{b}}$	0.218 ± 0.032	5.02 ± 2.26
RU	Phase I	0.213 ± 0.034	5.13 ± 2.38
RU	Phase I &II	0.208 ± 0.030	5.07 ± 2.30

Viral load is normalized by the reference virus PMMoV.

$$^{\textrm{a}}$$Phase I : 2023-01-08 to 2023-04-30.

$^{\textrm{b}}$Phase II: 2023-06-26 to 2023-10-01.

$$^{\textrm{c}}$$ 95% CI.

### Normalization by reference virus is more stable than normalization by volume flow

The number of gene copies in wastewater is not necessarily proportional to the amount of excreta from corona patients. Rain can dilute the concentration or heat can break down the RNA more quickly. Therefore, the measured number of gene copies must be skilfully normalized with the aid of flow rate, number of people in the catchment area or the concentration of a reference virus. The EU Commission recommends wastewater tests, but does not specify the exact method of normalization^32^. Our investigations for Rhineland–Palatinate indicate that normalization with the reference virus PMMoV is much more stable than normalizing by volume flow and people in the catchment area.

In Fig. 1 we have seen how well prevalence can be reconstructed from the viral load in wastewater if we normalize by PMMoV. Unfortunately, results become ambiguous if samples are normalized by volume flow and weighted by population. This is illustrated in Fig. 2. The prevalence in the SentiSurv cohort can be regarded as close to truth due to the high number of representative participants. The first wave at the end of February is still well reflected in the wastewater data. The new increase from mid-July onwards, however, leaves no corresponding traces in the wastewater, except for two spikes at the beginning of August and in mid-October. Table 3 illustrates the deviations of real measurements and the measurements to be expected from the adapted model. Viral loads are scaled back to incidences using the calibrated factors. Moreover, in order to have numbers the reader is used to, prevalence has been multiplied by 100,000. If the model fit is restricted to phase I of the SentiSurv study, which was in winter, then the quality of the wastewater fit is even better for normalization by volume flow than for normalization by reference virus (column MAE training data viral load (I)).

**Table 3:** Overview over the result quality in different configuration.

Scenario ID	MAE training data viral load (I)$^{\textrm{a}}$	MAE training data viral load (total)$^{\textrm{b}}$	MAE training data prevalence$^{\textrm{c}}$	Forecast Error prevalence $^{\textrm{d}}$
RP	**415**	310	276	127
RU	433	**265**	305	**94**
VP	466	440	245	430
VU	529	455	**241**	439

Best fit in bold face.

$^{\textrm{a}}$Mean absolute error of viral load (rescaled as prevalence) of data from 2023-01-09 to 2023-04-30 (phase I).

$^{\textrm{b}}$Mean absolute error of viral load (rescaled as prevalence) of data from 2023-01-09 to 2023-10-01 (phase I+II).

$^{\textrm{c}}$Mean absolute error of prevalence per 100,000 inhabitants from 2023-01-09 to 2023-04-30 (phase I).

$^{\textrm{d}}$Mean absolute error of prevalence per 100,000 inhabitants of data from 2023-06-26 to 2023-10-01 (phase II).

**Figure 3:**
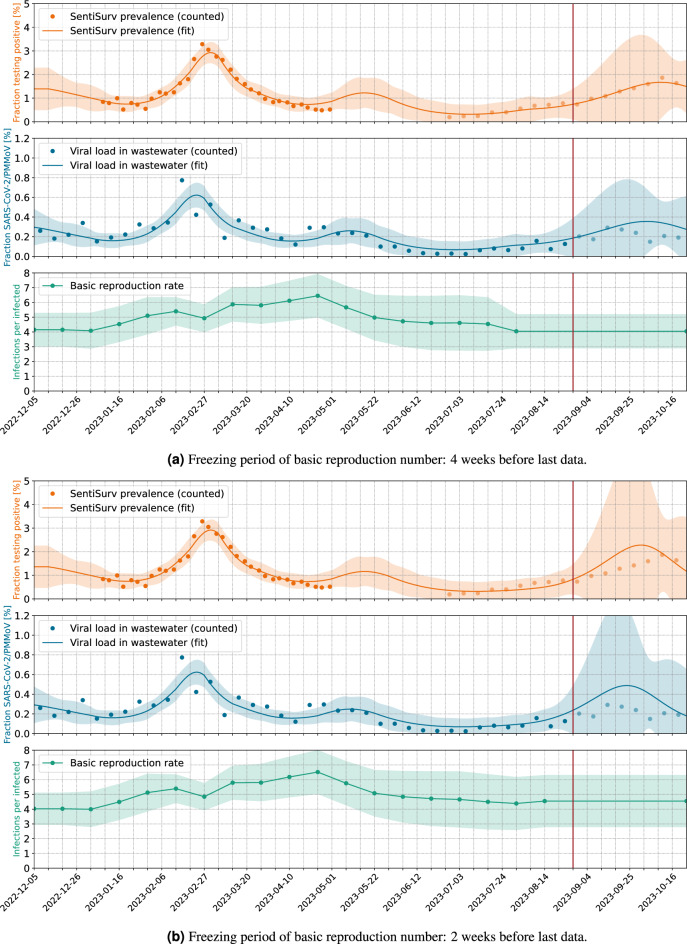
Short-term forecast of the prevalence in Rhineland–Palatinate for September and October 2023. Measurements plotted as pale dots do not enter the model fitting, but are used for validation. The vertical red lines mark the time of the forecast. Wastewater data are normalized by reference virus and uniform weighting of sites (RU).

### Short-term forecasts are possible

Our forecasts are based on the assumption that the basic reproduction number determined most recently will continue to be effective in the coming weeks. Over periods of up to 4 weeks, this approach has proven to be reasonably successful^33,34^. Over longer periods, however, too many uncertainties open up, e.g. unpredictable variant transitions. Figure 3a shows that not only the current prevalence can be derived from the wastewater, but that a short-term forecast of a few weeks is also possible. The pale dots again mark measurements that are not included in the model fit. So we consider the forecast that our model would have delivered at the end of August, based on the wastewater data accumulated up to that point and without any SentiSurv data from Phase II. We only consider the normalization with PMMoV here, which we have already identified as the more reliable one.

The quality of the forecast depends strongly on the modeling of the basic reproduction number, i.e. the reproduction rate in an environment of purely susceptible people. In the following, we will only call it the reproduction rate. We represent it as a piecewise linear function with grid points on a 2-week grid. The reproduction continues at a constant rate since a fixed period of time before the last available measurements. If this time is chosen too late, then this constant will be determined by only a few of the strongly fluctuating measurements of viral load. This can easily lead to over-fitting and nonsensical predictions, as can be seen in Fig. 3b. If, on the other hand, a sufficient number of measurements are allowed to determine the final reproduction rate, this results in a surprisingly good forecast of the prevalence for Rhineland–Palatinate in September and beginning of October 2023, see Fig. 3a. However, the correct choice of the freezing time is tricky. The earlier it is chosen, the better the noisy wastewater measurements average out. However, if the reproduction rate changes in reality, e.g. due to a change of variant, then this is not correctly captured by the model. In the case studied, a freezing period of 4 weeks proved to be effective.

## Limitations

One limitation of the study design is the observed infection dynamics. While having a considerable span of observations, we have only observed one complete infection wave, so far, which was monitored by both, waste water surveillance and cohort study. During a second minor wave in May, the SentiSurv study was unfortunately suspended, so that valuable references could not be established here. The third wave had not yet reached its peak at the end of the analysis period (first of October). Another temporal effect was observed in the summer, where the number of detected gene copies in some of the treatment sites were very close or even below the limit of detection. That had an effect in stabilizing the numbers, but may also bias the model in an unintended way

A second limitation originates from the way samples were collected. The weighting of wastewater samples would have been more straightforward if a homogeneous sample collection protocol could have been assumed. However, in order to follow a pragmatic, simple approach, some freedom has been given to the potential participants in the wastewater monitoring. Therefore, the location and configuration of the automatic samplers vary highly and are poorly documented. This might be the reason why the uniform sampling approach works better in our study than the population-based approach, as it is more robust against individual impure but large treatment sites.

Regarding the lab protocol, we can rule out one natural limitation since all wastewater samples have been analyzed by the same lab. That renders their values comparable. In a multi-lab setting, further normalization might be necessary.

Further biological limitations are induced by the variability of dominating virus variants. During the observation period, the dominant subvariants of Omicron, that were detected in the wastewater changed from BQ.1 to XBB.1.5, XBB1.9 and finally EG.5.1. Nevertheless, as shown, the identified relationship between the viral load and the prevalence turned out to be stable. We can however not guarantee this to hold for variants that undergo major changes from the current strains and show a significant change in the detectability in wastewaters.

With respect to the calibrating measurements from the cohort study, there are also limitations to mention. The SentiSurv data is used as true prevalence data in this study. However, with minors being excluded, there is a certain skew in the data. Due to a poor response rate of the contacted persons, the resulting distribution might suffer from a selection bias and is obviously not as representative as the original randomly chosen set has been. We did not do any statistical balancing for tourists and workers in the cities that contribute to the wastewater but are not eligible to participate in the prevalence study. When weighting the data of the selected cities to the total population of Rhineland–Palatinate, we did not include any demographic factors beyond population size. In some of the cities there is also not a perfect match between the city inhabitants and the population that is served by the respective treatment site. However, due to the high number of participants, our results indicate that the cohort composition is good enough to show reasonable prevalence data.

## Discussion

In the introduction, we highlighted the contributions of our study and discussed the existing approaches in the field of comparing wastewater viral loads with population-cohort based data. Building upon this foundation, it is important to emphasize the unique aspects of our work and how it complements and expands upon previous research. Our study differs from previous literature studies in two distinct cases. Firstly, some studies solely relied on officially reported case numbers without utilizing cohorts. In these cases, the correlation between wastewater viral loads and case numbers tended to be poor, making calibration challenging^10,11^. Secondly, other approaches also involved cohorts; however, it was either conducted in a highly restricted setting during the pandemic when campuses were locked down^18,19^ or involved a large effort and staff in testing^20^ In contrast, our study demonstrates the feasibility of calibration in a more deliberate setting. Nevertheless, achieving calibration in our setting requires significantly larger cohorts.

Our study has clearly demonstrated the strength of WBE methodologies, providing valuable insights into the dynamics of viral infections in wastewater and the role of the normalization technique. Nevertheless, it is important to acknowledge the mentioned limitations that may affect the transferability to other regions and time periods. The observed infection dynamics and the limited number of complete infection waves in our study highlight the need for further research to validate the predictive power of the model and assess its reliability in capturing the full extent of viral prevalence.

One notable finding of our study is the sensitivity of the results to the normalization method and weighting of different samples. It is still an open research question, what the gold standard for normalization is^10,21,29,35,36^ and without a proper reference cohort, it is hard to decide and may also be site-specific. Based on our data, the normalization with the PMMoV reference virus clearly outranks the flow based normalization, since we cannot see the beginning of the next wave that the SentiSurv data clearly indicates. We can only guess why flow normalization works fine in winter, but fails in summer. Maybe, temperature-dependent decay of viral RNA plays a role. The mean temperature of the collected wastewater was $10.9^{\circ }\hbox {C}$ from January to March 2023, but $16.4^{\circ }\hbox {C}$ from July to September 2023. Assuming that the RNA of SARS-CoV-2 and PMMoV have similar decay rates with temperature, their relation does not change along the way to the plant. However, the absolute number of gene copies of SARS-CoV-2 may decay quite differently depending on temperature. This could explain the different susceptibility of the normalization methods to heat. But this is a mere speculation and beyond our expertise. Future studies should aim to establish a robust and universally applicable normalization approach. While our study identified a stable relationship between viral load and prevalence, it is crucial to recognize that this relationship may not hold for variants that undergo major changes in detectability. Continued monitoring and adaptation of wastewater surveillance methods will be necessary to account for emerging variants.

All model parameters can be identified by fitting them to measured data. The fitting is done by solving an optimization problem. However, there seem to be different local minima, depending on the starting value for the protected. More precisely, different model dynamics can be found that reproduce the true measurements well and differ only moderately in prediction. This is because new infections increase with the model quantity *reproduction rate* and decrease with the model quantity *fraction of protected persons*. If both quantities are increased in a suitable way, then the observed number of new infections will stay the same. This limit to calibrability is discussed in more detail in “Calibration” section. For this paper, we have selected model dynamics that have base reproduction rates above 3, the value already found for the wild type. Automatically selecting the “correct” model dynamics will require further research.

The variation in wastewater sample collection protocols and the lack of standardized reporting methods pose challenges in data comparability and interpretation. Implementing uniform sampling protocols and internationally comparable reporting standards would enhance the reliability and usefulness of wastewater surveillance data^37^. Due to the smaller amount of data and hence worsened chances of convergence, we have not performed our analysis on the level of individual treatment plants versus the cohort data of the respective cities. With the data generally being available, it will be the subject of further research if and how the scaling factors vary between sites.

In summary, this gives a hopeful picture of transferability to other regions and time periods. No precise statistical correspondence is necessary to identify a quantitative correlation. It would therefore be conceivable, for example, to draw a cohort of similar size across Germany in order to calibrate the existing wastewater survey data from over 100 sewage treatment plants in Germany.

## Methods

The approach presented here aims to reconstruct the prevalence of SARS-CoV-2 in the population from wastewater data and to predict it several weeks ahead. For this purpose, the parameters of an epidemiological model are adjusted in such a way that two measured time series are well interpolated: the viral load in wastewater and the SARS-CoV-2 prevalence in a study cohort. In particular, we find a conversion factor between the two measured quantities. Even after the end of the cohort study, this factor makes it possible to infer the prevalence in the population by adjusting the model to the wastewater data only. The condition for the conversion factor to remain the same is that the amount of virus excreted by infectious persons does not change with the virus variant. This condition seems to be fulfilled for the variant changes of the year 2023. In order to predict prevalence over a few weeks, it is assumed that the transmission rate does not change from the last detected rate and simulation of the model is continued with this rate.

### Data sources

In the model presented here, in principle we only need two time series as input data sets. The first is the observed viral load of the wastewater treatment samples. The acquirement is a rather cost-efficient, but the values are not very telling and suffer from a high variation. The second source of information is the prevalence taken from a representative cohort. This data is more accurate, but quite expensive to retrieve. In the following, we will give more details on the source and processing of these two data sets.

#### Wastewater data

**Figure 4:**
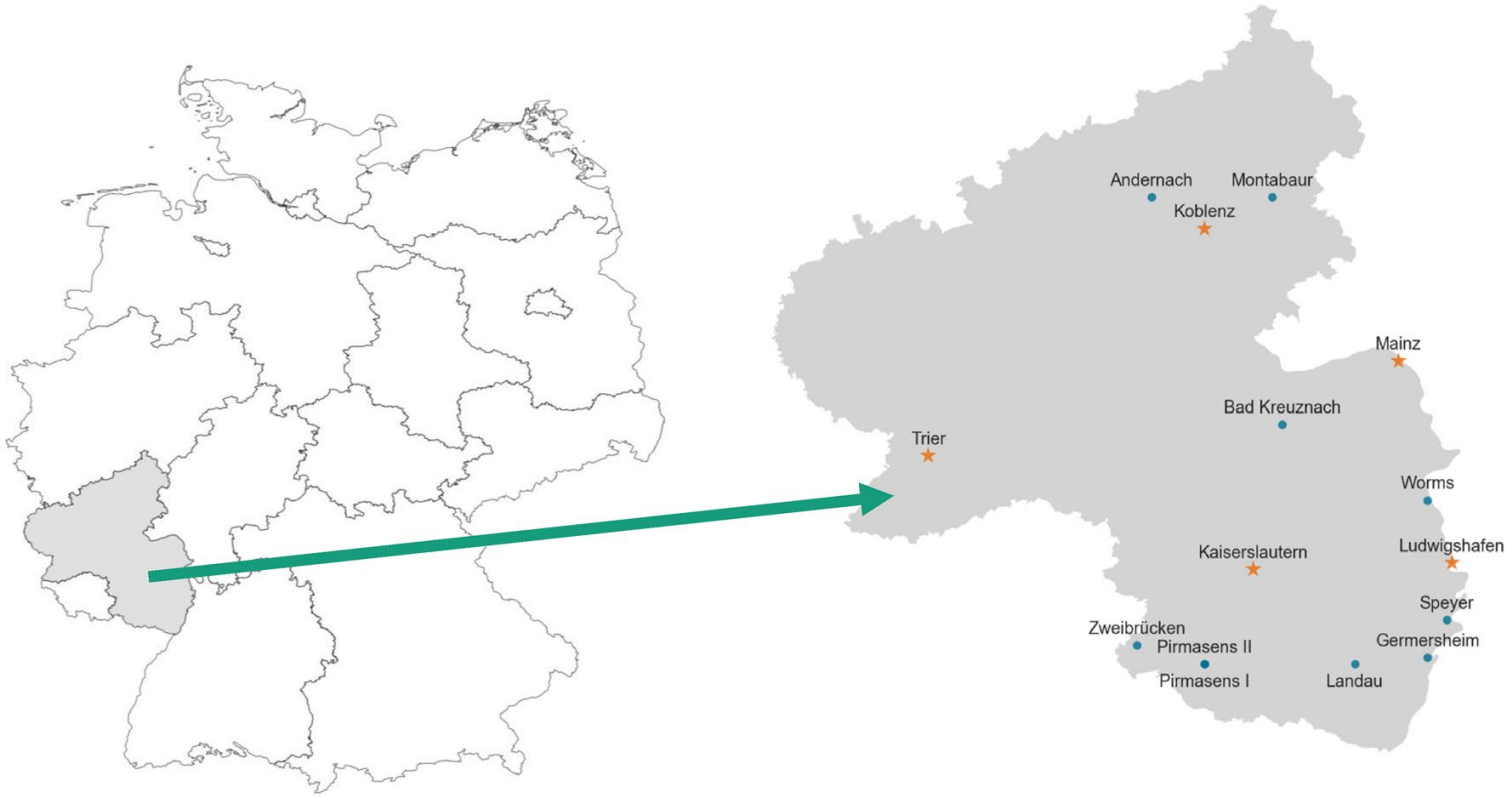
Locations of sampled waste water treatment plants (all markers) and locations of cities participating in the SentiSurv cohort (orange stars). The locations are distributed to cover uniformly population rather than area. On the left, the location of Rhineland–Palatinate in Germany is visualized.

The viral load concentration has been measured in 16 wastewater treatment sites in Rhineland–Palatinate. Their locations can be observed in Fig. 4. In Table 4, we compiled their static master data. Since October 2022, the participating treatment sites sample their water twice per week (in general Monday and Wednesday) in a 24-h composite sample. The sampling protocol allows for some flexibility in how and where the samples are taken. It is consistent with the guidelines which were developed for the EU project ESI-CorA^38^. Within the last months, modern automatic samplers were purchased and recommendations for volume- or flow proportionate sampling were given. Unfortunately, it is beyond our knowledge whether and who switched to this procedure and when. In each wastewater sample, the amount of *N*1 and *N*2 gene copies are being determined by the same commercial lab Bioscentia. For our analysis, we use the common approach of averaging *N*1 and *N*2 gene copies^39^. Additionally, the amount of reference virus PMMoV has also been measured. This is especially helpful for the comparison among wastewater treatment sites with different proportions of industrial wastewater. Another source of variance of viral load in wastewaters is the amount of rainfall that dilutes them. Therefore, the daily inflow is also measured and recorded and can be used for normalizing. Other parameters, such as the water temperature, the NH4-concentration or the pH-value have also been measured for most of the treatment sites. However, so far, they have not proven to be significant for the normalization of viral loads^1^. The executing laboratory Bioscentia followed the manufactor’s standard protocol for the Promega Maxwell ®RSC Enviro Total Nucleic Acid Kit for extracting the viral information.

**Figure 5:**
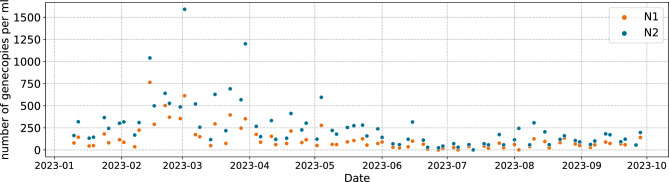
Raw data of N1 and N2 values for one treatment site (Kaiserslautern).

An exemplary time series from one site can be found in Fig. 5. It is easy to see that the measured values are subject to strong fluctuation. There are various reasons for that. The virus shedding rate varies already a lot among infected humans. The transportation through the sewage system and biological decay add further uncertainties, including discontinuous effects^13,14^. Especially with time proportionate samples, the collected water is not necessarily representative for the daily inflow of virus material^40^. Finally, the PCR method to determine the number of gene copies has a significant volatility itself^41^.

While it is thus impossible to detect a rise immediately, in the medium term, such patterns become obvious and share a high correlation with other measurements of infected people. A standard approach to smoothing this noisy data is the application of local regression techniques such as the LOESS method^42,43^. In this paper, as described in “Methods” section, we feed the original measurement values into a mathematical model that tries to fit a smooth functional relation as good as possible. It is therefore not necessary or advisable to smoothen the values beforehand.

**Table 4:** Characteristic data of the considered wastewater treatment sites.

City	Est. no. of connected population	Avg. dry daily flow rate $(\textrm{m}^3/\textrm{d})$ $^{\textrm{a}}$	Sample type$^{\textrm{a}}$	Sample location$^{\textrm{a}}$
Andernach	67,000	9000	Flow proportionate	Prior to sand filter
Bad Kreuznach	75,000	48,000	Time proportionate	Unspecified
Ludwigshafen$^{\textrm{b}}$	250,000	300,000	Time proportionate	After sand filter
Germersheim	28,000	4000	Time proportionate	Prior to sand filter
Kaiserslautern	140,000	26,000	Time proportionate	After sand filter
Koblenz	129,000	25,000	Time proportionate	After sand filter
Landau	55,000	11,000	Volume proportionate	After sand filter
Mainz	250,000	43,000	Time proportionate	After sand filter
Montabaur	25,000	9000	Time proportionate	After sand filter
Neustadt	64,000	10,000	Volume proportionate	After sand filter
Pirmasens I	27,000	6000	Volume proportionate	Prior to sand filter
Pirmasens II	15,000	3000	Volume proportionate	Prior to sand filter
Speyer	73,000	11,000	Unspecified	After sand filter
Trier	98,000	30,000	Time proportionate	Prior to sand filter
Worms	78,000	21,000	Time proportionate	After sand filter
Zweibrücken	36,000	9000	Volume proportionate	After sand filter

The cities in bold are the cities from which participants were drawn for the cohort.

$^{\textrm{a}}$As reported by wastewater treatment sites.

$^{\textrm{b}}$Ludwigshafen domestic wastewater is being treated in a large industrial treatment site.

#### Cohort study data

**Figure 6:**
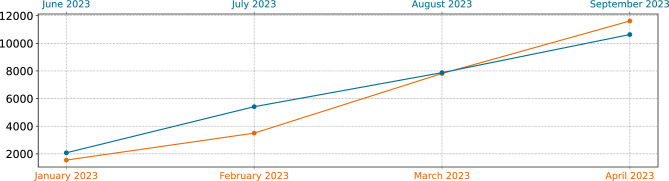
Median number of validly transmitted tests in SentiSurv cohort per month.

In late 2022, the federal state of Rhineland–Palatinate commissioned the University Medical Center of the Johannes Gutenberg-University Mainz to carry out a large longitudinal analysis on the prevalence of SARS-CoV-2 in the population, called SentiSurv^44,45^. The study was designed to last from December 2022 to the end of April 2023 and to include 14,000 participants testing themselves and reporting their status twice a week. However, recruitment got off to a slow start and a second study phase was added starting at the end of June, cf. Fig. 6. The participants were recruited through a letter sent to random addresses of adult residents of the cities Mainz, Ludwigshafen, Koblenz, Trier, and Kaiserslautern. These are the five largest cities in Rhineland–Palatinate and are well-distributed among the state which can be seen in Fig. 4. The participants initially carried out rapid tests twice a week on fixed days (Sunday and Wednesday) with specially sent, highly qualified self-tests as a nasal swab (SARS-CoV-2-Antigen Rapid Test Kit by VivaChek Biotech (Hangzhou) alias Verino®Pro, sensitivity of 99.13%, specificity of 100%). The test results are transmitted to the organisers via app. Most participants have skipped some tests. The first phase of data collection ended on April 30th. In late June, the data collection was revived and has been running since then. However, the frequency of testing has been reduced to once a week on a fixed day (Wednesday). With a super linear increase of included participants, phase 1 peaked with around 12, 000 validly transmitted tests. Phase 2 has by now a maximum of around 10, 000 validly transmitted tests. However, while phase 1 started in December 2022 with very few participants ($\ll 100$), in phase 2, many of the original participants could be recruited, therefore over 2000 valid tests were transmitted on the first test day. The median monthly number of valid, transmitted tests over time is visualized in Fig. 6 and the distribution is given in Table 5.

Throughout this manuscript, we use the term *prevalence* as the fraction of positively tested participants among all tested participants for one measurement of the SentiSurv study. In the context of the entire population of Rhineland–Palatinate, the term *prevalence* denotes the fraction of currently infected people relative to the total population.

**Table 5:** Characteristic data of the considered cities in the cohort study.

City	Official inhabitants$^{\textrm{a}}$^46^	Active participants$^{\textrm{b}}$		
		25%	50%	75%
Ludwigshafen	172,000	577	1228	1551
Kaiserslautern	99,000	644	1349	1862
Koblenz	114,000	424	970	1570
Mainz	218,000	930	1644	2335
Trier	111,000	566	1181	1594

$^{\textrm{a}}$Rounded for presentation only.

$^{\textrm{b}}$Quantiles based on valid transmitted tests in the timespan from 2023-01-08 to 2023-10-01.

### Epidemiological model

Virus propagation is modeled by a simple system of ordinary differential equations with state variables *newly infected* and *protected*. Our model is complex enough to capture the dominant effects of virus spread, yet simple enough to calibrate all parameters from the measured time series. The model is derived from a more detailed one with time delay^47^ by ignoring effects like vaccination, testing, and different age groups:

6 $$\begin{aligned} \dot{n}(t)&= \left( 1 - p(t) \right) \, \kappa (t) \, i(t) \end{aligned}$$

7 $$\begin{aligned} \dot{p}(t)&= \dot{n}(t) - \alpha \, p(t) \end{aligned}$$

8 $$\begin{aligned} i(t)&= \int \limits _{t-\tau _e}^{t-\tau _s} \dot{n}(\tau ) \, d\tau = n(t-\tau _s) - n(t-\tau _e) \; , \end{aligned}$$

where $t \in [0,T]$ indicates the time in days since the start of a chosen simulation period, $p(t) \in \left[ 0,1 \right]$ is the fraction of the selected population at time *t* who are protected against infection, *n*(*t*) is the number of infections since $t=0$ divided by the population, *i*(*t*) is the fraction of infectious persons at *t*, $\kappa (t)$ is the average number of transmitting contacts per day of an infectious person in a susceptible environment, and $\alpha$ is the waning rate of protection. The times $\tau _s$ and $\tau _e$ are start and end of the infectious phase, respectively, i.e. the original model assumes a constant emission of viruses during the infectious phase and no emission else.

The model contains delays, which requires initial values in form of function segments rather than numbers and may cause numerical instabilities. Therefore, we look for a purely differential equation with similar behavior and approximate $n(t+\Delta t)$ by its Taylor expansion of second order:

9 $$\begin{aligned} i(t)&= n(t-\tau _s) - n(t-\tau _e) \nonumber \\&\approx n(t) - \dot{n}(t) \, \tau _s + \frac{1}{2} \ddot{n}(t) \, \tau _s^2 - n(t) + \dot{n}(t) \, \tau _e - \frac{1}{2} \ddot{n}(t) \, \tau _e^2 \nonumber \\&= \left( \tau _e - \tau _s \right) \left[ \dot{n}(t) - \frac{\tau _e + \tau _s}{2} \ddot{n}(t) \right] \, . \end{aligned}$$

With central time of the infectious phase $\tau$, infectious period $\delta$, and the fraction of newly infected persons per day *v*(*t*) we find

10 $$\begin{aligned} i(t)&= \delta \left( v(t) - \tau \, \dot{v}(t) \right) \end{aligned}$$

with

11 $$\begin{aligned} \delta = \tau _e-\tau _s \, , \quad \tau = \frac{\tau _e + \tau _s}{2} \, , \quad v(t) = \dot{n}(t). \end{aligned}$$

Replacing (10) in (6) and solving for $\dot{v}$ gives the following simplified set of differential equations:

12 $$\begin{aligned} \dot{v}(t)&= \frac{1}{\tau } \left( 1 - \frac{1}{r(t) \left( 1-p(t) \right) } \right) v(t) \end{aligned}$$

13 $$\begin{aligned} \dot{p}(t)&= v(t) - \alpha \, p(t) \end{aligned}$$

with basic reproduction rate

14 $$\begin{aligned} r(t) = \delta \, \kappa (t). \end{aligned}$$

The central time of the infectious phase $\tau$ is closely related to the *serial interval* of a disease and, for convenience, we will use this term for $\tau$. Note that vaccination can easily be included in the model, if needed, by adding a vaccination rate to the right-hand side of Eq. (13). The reader may wonder why we have not used a standard SEIR model. There are two reasons for this. Firstly, a SEIR model requires initial values to be fitted for three dynamic state variables instead of just two, as in our case. This modification stabilizes parameter fitting considerably. Secondly, we want to keep the model compatible with a detailed model, which includes additional effects such as testing or variant transitions and which we have already used successfully to create forecasts for other institutions^33,34^.

#### Stationary solutions

For a given constant reproduction rate $\bar{r}$ we have two stationary solutions: the trivial one, which is asymptotically stable for $\bar{r}<1$ and unstable for $\bar{r}>1$:

15 $$\begin{aligned} p = 0 \, , \quad v = 0\, , \end{aligned}$$

and the non-trivial one:

16 $$\begin{aligned} \bar{p} = 1 - \frac{1}{\bar{r}} \, , \quad \bar{v} = \alpha \, \bar{p}. \end{aligned}$$

The latter exists only for $\bar{r} > 1$, where it is asymptotically stable. The stability analysis can be found in the Supplementary Information 3.

### Link to measurements

**Figure 7:**
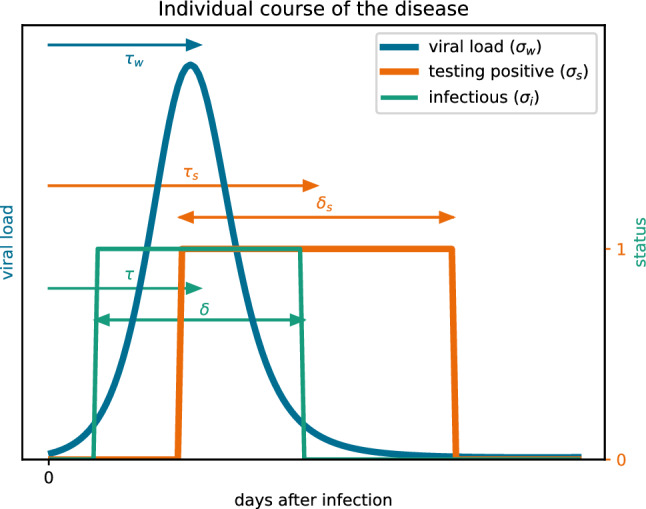
Schematic representation of the integration kernels for viral load, proportion of infectious persons and proportion of persons testing positive. Note that the periods of shedding the virus, being infectious and testing positive are not identical. In particular, antigen tests are triggered only a few days after a person has become infectious, but may show a positive result even if the viral load is no longer sufficient for infection^48^.

In this section, we relate the two measurable variables, *prevalence of people testing positive* and *viral load in the wastewater*, to our model variable *i*, i.e. the *proportion of infectious persons*. We will see that, in principle, *i* cannot be completely reconstructed from the measured variables, but can only be determined down to a scaling factor and a time offset. We choose these parameters in a plausible, yet arbitrary way. Therefore, the resulting parameters for offset and scaling should not be over-interpreted virologically. They are simply numerical parameters with which the measurable variables *prevalence* and *viral load* can best be reproduced from the invisible model variable *i* within the investigated period. We now show how prevalence, viral load and the proportion of infected persons are structurally linked.

Let *s*(*t*) be the fraction of persons testing positive at time *t* according to the SentiSurv study and *w*(*t*) the mean viral load excreted per person and day at time *t*. Moreover, let *i*(*t*) and $\nu (t)$ be the fraction of infectious and newly infected persons, respectively, described in our model. We assume that the net effect of all the individual courses of the disease is the same as if all people behave like a suitable standard person after infection. In this case, there are integration kernels $\sigma _*$ such that:

17 $$\begin{aligned} w(t) = \int \limits _{-\infty }^t \sigma _w(t-t') \, \nu (t') \, dt' \, , \quad s(t)&= \int \limits _{-\infty }^t \sigma _s(t-t') \, \nu (t') \, dt' \, , \quad i(t) = \int \limits _{-\infty }^t \sigma _i(t-t') \, \nu (t') \, dt'. \end{aligned}$$

Qualitative plots of the kernels are depicted in Fig. 7. The kernel $\sigma _w$ of viral load is typically shaped like an asymmetric bell. It is proportional to the viral load produced by an individual *t* days after infection. Let $t_{\textrm{max}}$ be the time after infection when the individual viral load is maximal. Then people who were infected at time $t' = t-t_{\textrm{max}}$ contribute most to the integral representing *w*.

However, the kernel $\sigma _s$ which generates the SentiSurv data is a box. This is because all people testing positive contribute uniformly to the integral representing *s*, regardless of whether the individual viral load is currently at maximum or moderate. The same holds true for $\sigma _i$ generating the fraction of infectious persons. However, the box is usually shifted towards the start of infection. This is because people start being infectious slightly before antigen tests are triggered and tests remain positive even if only dead viral material is left in the nasal cavity.

Typically, details of the integration kernels $\sigma _*$ are not available. However, if the length of $\sigma _*$ is short, then the fraction of newly infected persons $\nu$ may be approximated by its linearization, which gives for some quantity $x \in \left\{ w,s,i \right\}$:

18 $$\begin{aligned} x(t)&= \int \limits _{-\infty }^t \sigma _x (t-t') \, \nu (t') \, dt' \; \approx \int \limits _{-\infty }^t \sigma _x (t-t') \, \left( \nu (t) + \dot{\nu }(t) (t' - t) \right) \, dt' \nonumber \\&= \nu (t) \underbrace{\int \limits _{0}^{\infty } \sigma _x(t'') \, dt''}_{\delta _x} - \dot{\nu }(t) \underbrace{\int \limits _{0}^{\infty } \sigma _x(t'') \, t'' \, dt''}_{\delta _x \tau _x} \; = \delta _x \left( \nu (t) - \tau _x \, \dot{\nu }(t) \right). \end{aligned}$$

For *i* we skip the index of $\delta$ and $\tau$ and find again Eq. (10). Now we express *w* and *s* as scaled and shifted versions of *i*. Once more, we assume locally linear behavior of $\nu$, i.e. $\nu (t+h) \approx \nu (t) + h \, \dot{\nu }(t)$, $\dot{\nu }(t+h) \approx \dot{\nu }(t)$. After some algebra we finally get

19 $$\begin{aligned} w(t) \approx \frac{\delta _w}{\delta } \, i\left( t+ (\tau -\tau _w) \right) \, , \quad s(t) \approx \frac{\delta _s}{\delta } \, i\left( t+ (\tau -\tau _s) \right) \, . \end{aligned}$$

As we can only observe *w* and *s*, it is impossible to uniquely identify the parameters $\delta$ and $\tau$ of the hidden model variable *i*. This can be seen as follows. Assume that the true dynamics of the model were shifted one day towards the past. Increasing also the shifts $\tau _w$ and $\tau _s$ by one day would then lead to exactly the same measurements, i.e. the shifts cannot be fully identified from the available measurements. Therefore, we specify that *i* is aligned with the viral load *w*, i.e. $\tau \equiv \tau _w$. Note that this step does not induce any errors in the forecasting, but just changes slightly the interpretation of the hidden model variable *i*. Next we have a look at the scaling factors. Let us come back to the model, i.e. Eqs. (10), (12), and (13). We observe that *i* is just a post processing variable not entering the dynamics and the only role of $\delta$ is to get from the newly infected persons $\nu$ to the infectious ones *i*. If $\delta$ is doubled and so are $\delta _s$ and $\delta _w$, then we have exactly the same model dynamics and the same measurements. We fix this ambiguity by identifying the scaling factors of people being infected and people testing positive, i.e. $\delta \equiv \delta _s$. Once more, only the interpretation of the hidden variable *i* will slightly change. Finally, we have

20 $$\begin{aligned} w(t)&\approx \frac{\delta _w}{\delta _s} \, i(t) =: \Gamma \, i(t) \end{aligned}$$

21 $$\begin{aligned} s(t)&\approx i\left( t - (\tau _s-\tau _w) \right) =: i\left( t-\Delta t \right) \end{aligned}$$

with identifiable parameters $\Gamma$ and $\Delta t$.

**Table 6:** Quantities of the SentiSurv study aggregated over age groups and cities in order to compute the prevalence of SARS-CoV-2 in Rhineland–Palatinate.

Symbol	Definition	Min	Mean	Max
*t*	Testing date (twice per week until 2023-04-30, once a week since 2023-06-28)	2023-01-08	–	2023-09-27
$N_v(t)$	Number of participants in the SentiSurv study who have currently submitted a valid test	166	6747	12,198
$N_p(t)$	Number of participants in the SentiSurv study currently testing positive	4	72	166

So far, we have derived a structural model of how the fraction of people testing positive *s* and the viral load in the wastewater *w* are related to the fraction of infectious people *i*. Now we link the variables *w* and *s* to actual measurements.

We first consider the time series of the SentiSurv study. From the aggregated data provided by the University Medical Center Mainz, we use the aggregated quantities listed in Table 6 to construct an estimate of the prevalence of SARS-CoV-2 in the population of Rhineland–Palatinate, i.e. the fraction of people who are currently tested positive.

22 $$\begin{aligned} s(t)&= \frac{N_p(t)}{N_v(t)}. \end{aligned}$$

**Table 7:** Quantities measured at sewage treatment plants used to compute the viral load.

Symbol	Definition	Typical value	Unit
*N*1	Copies of first SARS-CoV-2 gene sequence per sample volume	200	ml$^{-1}$
*N*2	Copies of second SARS-CoV-2 gene sequence per sample volume	400	ml$^{-1}$
$\overline{N}$	Average number of SARS-CoV-2 gene copies per sample volume: $\frac{N_1+N_2}{2}$	300	ml$^{-1}$
*R*	Ratio of mean value of SARS-CoV-2 gene copies and gene copies of the reference virus PMMoV multiplied by 100,000	200	–
*Q*	Volume of wastewater passing a sewage treatment plant per day	40,000	m$^3$/d
*P*	Number of persons connected to a sewage treatment plant	90,000	–

Now we turn to the wastewater samples. In order to compute normalized viral loads for Rhineland–Palatinate we use the measured quantities listed in Table 7. Note that the quantity belonging to treatment plant *j* will be subscripted accordingly, i.e. $P_j$ represents the number of persons connected to treatment plant *j*. $R_j(t)$ is the ratio of gene copies of SARS-CoV-2 and PMMoV measured at site *j* at day *t*. If there is no measurement available at that day, the time series is filled with 0. The same notation holds for the other measurements. We take averages over the valid measurements of all plants and of all days of a certain week. The sites are weighted either equally or according to the connected population. Normalized viral loads of the wastewater are denoted by *w*. First subscript *R* indicates normalization by reference virus and first subscript *V* indicates normalization by volume flow. The second subscript indicates the weighting. *P* stands for population and *U* for uniform (cf. Table 1). The mean value of week *k* is assigned to its Thursday $t_k$. We compare the following normalization schemes, where *S* denotes the set of sites, $D_k$ the set of days of week *k*, and $\chi$ is the characteristic function $$\chi (x) = \left\{ \begin{array}{cc} 0 &{} x\le 0 \\ 1 &{} x>0 \end{array} \right.$$ . Then the differently normalized viral loads in wastewater read

23 $$\begin{aligned} w_{RP}(t_k)&= \frac{\sum \limits _{j \in S} \, \sum \limits _{t \in D_k} P_j\, R_j(t)}{\sum \limits _{j \in S} \, \sum \limits _{t \in D_k} P_j \, \chi \big (R_j(t)\big )} \end{aligned}$$

24 $$\begin{aligned} w_{RU}(t_k)&= \frac{\sum \limits _{j \in S} \, \sum \limits _{t \in D_k} R_j(t)}{\sum \limits _{j \in S} \, \sum \limits _{t \in D_k} \chi \big (R_j(t)\big )} \end{aligned}$$

25 $$\begin{aligned} w_{VP}(t_k)&= \frac{ \sum \limits _{j \in S} \, \sum \limits _{t \in D_k} \overline{N}_j(t) \, Q_j(t)}{\sum \limits _{j \in S} \, \sum \limits _{t \in D_k} P_j \, \chi \big (\overline{N}_j(t) \, Q_j(t) / P_j\big )} \end{aligned}$$

26 $$\begin{aligned} w_{VU}(t_k)&= \frac{ \sum \limits _{j \in S} \, \sum \limits _{t \in D_k} \overline{N}_j(t) \, Q_j(t) / P_j}{\sum \limits _{j \in S} \, \sum \limits _{t \in D_k} \chi \big (\overline{N}_j(t) \, Q_j(t) / P_j\big )}. \end{aligned}$$

How are these estimators related to the fraction of infectious people of our model? Let $\Gamma$ be the number of SARS-CoV-2 gene copies that an infectious person excretes on average per day, $\Gamma _{\text {PMMoV}}$ the corresponding rate of the reference virus, and

27 $$\begin{aligned} \gamma&= \Gamma / \Gamma _{\text {PMMoV}}. \end{aligned}$$

Normalizing by the reference virus, we assume that the fraction of infectious people is the same all over Rhineland–Palatinate and within a given week. Moreover, we assume that the two virus types decay at the same rate along their way to the plant. Then we find

28 $$\begin{aligned} R_j(t)&= \frac{\Gamma \; i_j(t)\, P_j}{\Gamma _{\text {PMMoV}}\, P_j} = \gamma \; i(t_k)\quad \text {for} \; t \in D_k\, , \end{aligned}$$

which gives

29 $$\begin{aligned} w_{RP}(t_k)&= \frac{\sum \limits _{j \in S} \, \sum \limits _{t \in D_k} P_j\, \gamma \; i(t_k)}{\sum \limits _{j \in S} \, \sum \limits _{t \in D_k} P_j \, \chi \big (\gamma \; i(t_k) \big )} = \gamma \; i(t_k) \end{aligned}$$

30 $$\begin{aligned} w_{RU}(t_k)&= \frac{\sum \limits _{j \in S} \, \sum \limits _{t \in D_k} \gamma \; i(t_k)}{\sum \limits _{j \in S} \, \sum \limits _{t \in D_k} \, \chi \big (\gamma \; i(t_k) \big )} = \gamma \; i(t_k). \end{aligned}$$

Normalizing by volume flow, we have to make further assumptions: the samples are drawn in a representative way and the gene copies are stable on their way to the sewage treatment plant. Then we have

31 $$\begin{aligned} \overline{N}_j(t)&= \frac{\, \Gamma \; i_j(t) \, P_j}{Q_j(t)} = \frac{\, \Gamma \; i(t_k) \, P_j}{Q_j(t)} \quad \text {for} \; t \in D_k . \end{aligned}$$

This gives again identical relations of the viral load to the infectious fraction for both weightings:

32 $$\begin{aligned} w_{VP}(t_k)&= \frac{\sum \nolimits _{j \in S} \, \sum \nolimits _{t \in D_k} \Gamma \; i(t_k) \, P_j \, \chi \big ( Q_j(t) \big )}{\sum \nolimits _{j \in S} \, \sum \nolimits _{t \in D_k} P_j \, \chi \big ( \Gamma \; i(t_k) \, P_j \, \chi (Q_j(t)) \big )} = \Gamma \; i(t_k) \end{aligned}$$

33 $$\begin{aligned} w_{VU}(t_k)&= \frac{\sum \nolimits _{j \in S} \, \sum \nolimits _{t \in D_k} \Gamma \; i(t_k) \chi \big ( Q_j(t) \big )}{\sum \nolimits _{j \in S} \, \sum \nolimits _{t \in D_k} \chi \big ( \Gamma \; i(t_k) \, \chi (Q_j(t)) \big )} = \Gamma \; i(t_k). \end{aligned}$$

This means that all normalization schemes provide viral loads which are proportional to the model state *i*, the fraction of infectious people. The proportionality constant $\gamma$ is valid for normalization by reference virus and the proportionality constant $\Gamma$ is valid for normalization by volume flow.

**Figure 8:**
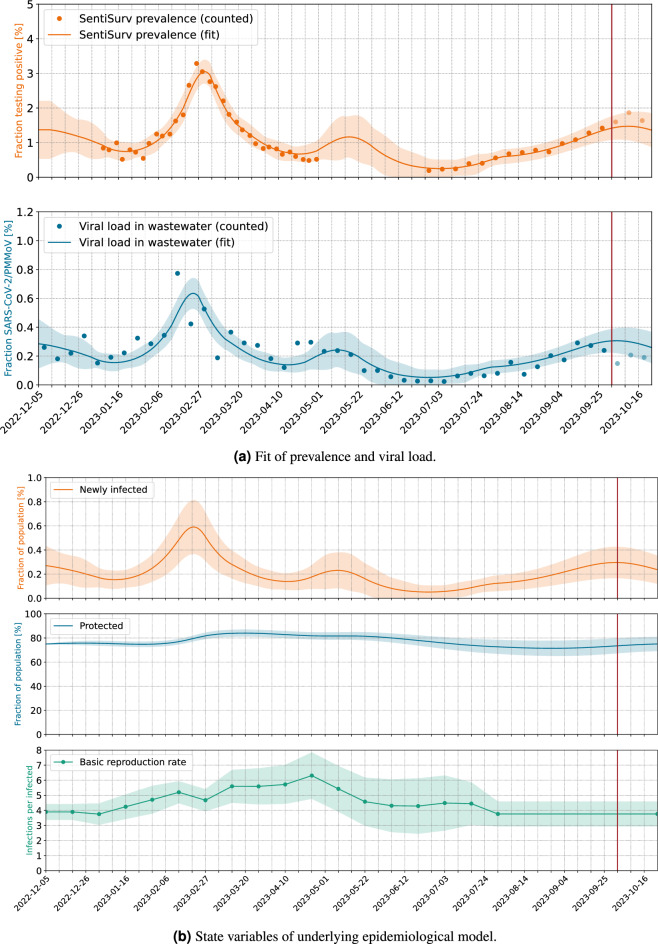
Reference calibration.

**Figure 9:**
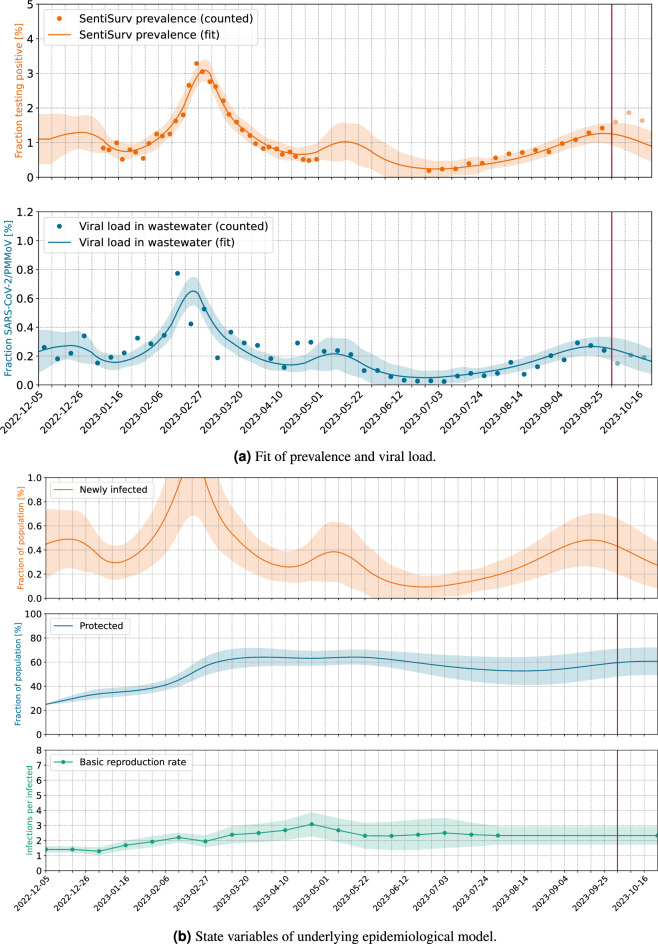
Calibration with low initial value of protected people.

### Calibration

The state variables of the model are not directly measurable. In order to predict the course of the epidemic, we proceed as follows.

Starting with a plausible initial guess of the model parameters we simulate our model, i.e. Eqs. (12), (13), (10), and synthesize the quantities we can measure, the viral load in the wastewater and the people within the SentiSurv cohort currently testing positive. Here, we use Eqs. (29)–(33). The model parameters are then adjusted such that the deviation between synthetic and true measured values becomes minimal. More precisely, the maximum likelihood estimator of the parameters is determined automatically via non-linear optimization. In doing so, we take advantage of the fact that our simulator is capable of automatic differentiation: if certain parameters are adjusted, the simulation not only provides a deviation between synthetic and actual measurements, but also how these deviations change in the case of small perturbations of the parameters. This knowledge significantly speeds up the optimization. Moreover, it enables us to derive prediction errors from measurement errors.

Constraints, e.g. positivity of the reproduction rate, are implemented as soft constraints, i.e. violations are penalized in the objective function. The result of the parameter identification depends on which measurement errors are assumed for viral load and prevalence. Since no reliable information is available here, self-consistency is aimed for, i.e. after a first parameter identification, an empirical standard deviation between measured and synthesized measurements is computed for each of the two measured quantities and this is used as a measurement error for a the next parameter identification. After a few iterations, the assumed and empirical standard deviation have usually converged. Further details on the parameter identification we use can be found in the Supplementary Information 3.

The software consists of a fast computational core programmed in C++ and a Python interface through which scenarios can easily be built and modified. Data is exchanged via configuration and result files in JSON format.

A special feature is that the role of a variable in the configuration file can be easily changed between constant, measured value, and unknown. In particular, it is possible to specify which measurements are to be included in the parameter fit and which are not.

We close this article by a sensitivity analysis of our model fit. We have chosen our epidemiological model to be as simple as possible so that all parameters can be identified from measurements. Nevertheless, some arbitrary settings remain to be chosen: the type of normalization of wastewater data, the phases of SentiSurv considered in a fit, the duration over which the reproduction rate is frozen at the end of a fit, and the initial proportion of protected people. The results of the sensitivity study are given in Table 8.

**Table 8:** Sensitivity analysis of fitted model parameters.

Parameter$^{\textrm{a}}$	Reference$^{\textrm{b}}$	Normalization RP	Fit end 2023-05-01	Initial protected fraction 0.25	Freezing period 4 weeks
$\alpha$	0.003 ± 0.001	0.003 ± 0.001	0.003 ± 0.001	0.005 ± 0.004	0.002 ± 0.001
$\delta$	4.93 ± 1.83	4.84 ± 1.84	4.94 ± 1.84	2.60 ± 1.02	5.24 ± 1.78
$\tau$	3.58 ± 2.04	3.54 ± 2.01	3.67 ± 2.09	3.77 ± 2.00	3.70 ± 2.06
$v_0$	0.003 ± 0.002	0.003 ± 0.002	0.003 ± 0.002	0.004 ± 0.003	0.002 ± 0.001
$r_{\text {min}}$	3.752 ± 0.697	3.749 ± 0.710	3.750 ± 0.849	1.293 ± 0.243	3.783 ± 0.730
$\bar{r}$	4.765 ± 1.181	4.768 ± 1.182	4.768 ± 1.246	2.238 ± 0.599	4.795 ± 1.199
$r_{\text {max}}$	6.308 ± 1.524	6.172 ± 1.542	6.272 ± 1.532	3.083 ± 0.772	6.432 ± 1.520
$\gamma$	0.208 ± 0.031	0.218 ± 0.033	0.213 ± 0.035	0.210 ± 0.028	0.209 ± 0.031
$\Delta$	5.07 ± 2.30	5.02 ± 2.27	5.13 ± 2.38	5.11 ± 2.09	5.05 ± 2.30

$^{\textrm{a}}$Parameters are $\alpha$: protection loss rate [1/d], $\delta$: infectious period [d], $\tau$: serial interval [d], $\nu _0$: fraction of newly infected people at start, $r_\text {min}$, $\bar{r}$, $r_\text {max}$: minimal, average, maximal reproduction rate, $\gamma$: fraction of gene copies of SARS-CoV-2 and PMMoV, $\Delta$: offset of prevalence to viral load [d].

$^{\textrm{b}}$Reference configuration: normalization RU, fit end: 2023-10-01, initial fraction of protected people: 0.75, freezing period: 8 weeks. Single parameters are modified with respect to the reference. Ranges are 95% CI’s.

Both, the infectious period $\delta$ and the serial interval $\tau$ of the reference configuration correspond well with the experimental findings in^48^, where the expected infectious period is given as 1.6+2.7 = 4.3 days and the RNA viral load growth phase as 3.6 days. Note that we have found similar parameters just from fitting an epidemiological model without applying individual PCR tests or enumerating cultivable virus.

The most interesting parameters for linking prevalence and the viral load in wastewater, $\gamma$ and $\Delta$, are very robust against any changes in the calibration setup. However, the long offset $\Delta$ between viral load and prevalence of about 5 days needs some discussion. The prevalence refers to the fraction of people who would generate a positive antigen test. As illustrated in Fig. 7, this is not identical to being infectious. It is known that antigen tests are triggered only a few days after a person has become infectious^48^. Later, the viral shedding becomes too small to induce new infections, while sensitive antigen tests still show a positive result. Moreover, people with symptoms usually isolate themselves, which further reduces the effective infectious phase, even if the viral load is still critical. Therefore, we can expect a positive delay between viral load and prevalence. But even if the phase of positive tests starts only after the serial interval, it must last 10 days to have a $\Delta$ of 5 days. According to our experience from public test centers this happens quite often, but not on average. This may indicate that the true value of the offset is rather in the lower part of the 95% confidence interval $\Delta \in [2.77\, , \, 7.37]$. Note that the estimate of $\Delta$ is quite inaccurate. In first place, $\Delta$ is a numerical parameter to match the measured time series of viral load and prevalence in an optimal way, which should not be over-interpreted virologically.

Moreover, we observe that all parameter estimates are almost the same, independent of the normalization method and the fact that phase II of the SentiSurv study is included in the fit. The latter means that the basic parameters of SARS-CoV-2 were quite stable within the analyzed period from December 2022 to October 2023. Note that no major variant changes were observed and neither the antigen test kits nor the protocol for wastewater analysis were modified in this period.

Other changes to the configuration have a greater impact. A too short freezing time changes slightly the estimates of protection loss rate, infectious period, and initial fraction of newly infected persons.

The biggest influence is due to the initial number of protected persons. Note that this number cannot be reconstructed from the measurements: the number of new infections in Eqs. (12) and (13) is determined by the measured values for prevalence and viral load. Equation (13) then yields a different curve for *p* (protected) for each starting value $p_0$. Equation (12) can then be solved for the reproduction rate *r*. I.e. for each initial value $p_0$, curves can be constructed for *p* and *r* that lead to the same new infections $\nu$ and thus to the same measurements. However, a different choice of the initial fraction of protected people yields a slightly different forecast, which is important in political consulting, cf. Figs. 8 and 9.

Throughout this article, we have chosen a starting value of $p_0$=0.75, since the reproduction rates then lie above the reproduction rate of the wild type ($r=\text {3.38} \pm \text {1.40}$^49^). Moreover, the initial fraction of protected people is in the same range as later in the year.

### Supplementary Information

Supplementary Information 1.

Supplementary Information 2.

Supplementary Information 3.

## Data Availability

The absolute viral load in the wastewater of 16 sites in Rhineland–Palatinate is published by the Landesuntersuchungsamt Rheinland-Pfalz. Values normalized by the volume flow are published by the Robert Koch Institut. The complete data set including volume flow and the viral load normalized by PMMoV is attached as Supplementary Information 2 to the Supplementary Material with permission of W. Lehnen, Ministry of Science and Health Rhineland–Palatinate. Data on the cohort study *SentiSurv* is published by the University Medical Center of the Johannes Gutenberg University Mainz and, for convenience, is attached as Supplementary Information 1 to the Supplementary Material with permission of Prof. Dr. med. P. Wild, Johannes Gutenberg University Mainz.
