# Supplementary material for: Estimating the COVID-19 prevalence from wastewater
Source: Sci Rep. 2024 Jun 22;14:14384. doi: 10.1038/s41598-024-64864-1 (PMC11193770; doi:10.1038/s41598-024-64864-1)
Supplement: Supplementary file 3 — Supplementary Information 3. [file 41598_2024_64864_MOESM3_ESM.pdf]

Appendix to
Estimating the Covid-19 Prevalence from Wastewater

Jan Mohring[1,*], Neele Leithäuser[1], Jarosław Wlazło[1], Marvin Schulte[1], Maximilian Pilz[1], Johanna Münch[1], and Karl-Heinz Küfer[1]

[1]Fraunhofer Institute for Industrial Mathematics, Kaiserslautern, 67663, Germany
[*]jan.mohring@itwm.fraunhofer.de

ABSTRACT

This Appendix includes a stability analysis of the epidemiological model and an overview of our approach to parameter identification, which is used to fit the model to measured values of viral load and prevalence. This information has been separated from the main article as the content may be too mathematical for most readers.

Stability analysis

Recall the system of ordinary differential equations (8)-(9) of the main article with constant reproduction rate $\bar{r}$,

$$\dot{v}(t) = \frac{1}{\tau}\left(1 - \frac{1}{\bar{r}\,(1-p(t))}\right) v(t) \tag{1}$$

$$\dot{p}(t) = v(t) - \alpha\, p(t)\;, \tag{2}$$

which has the stationary solutions

$$p = 0\,, \quad v = 0\,, \tag{3}$$

and

$$\bar{p} = 1 - \frac{1}{\bar{r}}\,, \quad \bar{v} = \alpha\,\bar{p}\;. \tag{4}$$

In order to prove their stability depending on $\bar{r}$, we linearize the system at the fixed points. Therefore, we compute

$$\frac{\partial \dot{v}(t)}{\partial v} = \frac{1}{\tau}\left(1 - \frac{1}{\bar{r}\,(1-p(t))}\right), \tag{5}$$

$$\frac{\partial \dot{v}(t)}{\partial p} = -\frac{v(t)}{\tau\bar{r}\,(1-p(t))^2}, \tag{6}$$

$$\frac{\partial \dot{p}(t)}{\partial v} = 1, \tag{7}$$

$$\frac{\partial \dot{p}(t)}{\partial p} = -\alpha. \tag{8}$$

Introducing the state vector $x(t) = \begin{bmatrix} v(t) \\ p(t) \end{bmatrix}$, the linearized system around $x = \begin{bmatrix} 0 \\ 0 \end{bmatrix}$ gives

$$\dot{x}(t) = \underbrace{\begin{bmatrix} \frac{1}{\tau}\left(1-\frac{1}{\bar{r}}\right) & 0 \\ 1 & -\alpha \end{bmatrix}}_{=:\mathrm{A}} x(t). \tag{9}$$

The spectrum of A reads

$$\sigma\left(\mathrm{A}\right) = \left\{ \frac{1}{\tau}\left(1 - \frac{1}{\bar{r}}\right), -\alpha \right\}. \tag{10}$$

Both eigenvalues are real and negative for $\bar{r} < 1$, hence the stationary solution $x = \begin{bmatrix} 0 \\ 0 \end{bmatrix}$ is asymptotically stable for the linearized system. By Hartman-Grobman theorem[1], this result can be transferred to the nonlinear system.

For $\bar{r} > 1$, we have

$$-\alpha < 0 < \frac{1}{\tau}\left(1 - \frac{1}{\bar{r}}\right) \tag{11}$$

and hence $x = \begin{bmatrix} 0 \\ 0 \end{bmatrix}$ is a saddle (thus unstable) in the linear as well as the nonlinear case. The stable subspace in both cases corresponds to solutions with $v(t) = 0$ as one can easily verify.

The linearized system at the non-trivial fixed point $\bar{x} = \begin{bmatrix} \bar{v} \\ \bar{p} \end{bmatrix}$ is given by

$$\dot{x}(t) = \underbrace{\begin{pmatrix} 0 & -\frac{\alpha(\bar{r}-1)}{\tau} \\ 1 & -\alpha \end{pmatrix}}_{=:\mathrm{B}} (x(t) - \bar{x})\,. \tag{12}$$

Computing the characteristic polynomial of B

$$\chi_{\mathrm{B}}(\lambda) = \lambda(\lambda + \alpha) + \frac{\alpha(\bar{r}-1)}{\tau} \tag{13}$$

we find its eigenvalues

$$\lambda_{\pm} = -\frac{\alpha}{2} \pm \sqrt{\left(\frac{\alpha}{2}\right)^2 - \frac{\alpha(\bar{r}-1)}{\tau}}. \tag{14}$$

A biologically meaningful stationary solution with $p > 0$ exists only if $\bar{r} > 1$. As both, α and τ, are positive, the root term in Equation (14) is either smaller $\frac{\alpha}{2}$ or imaginary. Therefore, both eigenvalues have negative real part and we can conclude asymptotic stability of $\bar{x}$ in the linear and the nonlinear case.

Parameter identification

In this section we formulate a general calibration problem that can be solved with our software AD-Ident. We explain the main steps in the solution process and describe the implementation and interface principles. Finally, we state the task of estimating the Covid-19 incidence from wastewater as an instance of the defined optimization problem, which leads to the results shown above.

General model and assumptions

Let $\theta \in \Theta \subset \mathbb{R}^{n_p}$ represent the model parameters and $\mathscr{R}$ be a certain class of functions over the interval $[t_0, +\infty)$, for a given initial time t_0. Furthermore, let $x \in \mathscr{R}^{n_x}$ denote a vector of state variables and $y \in \mathscr{R}^{n_y}$ be a vector of outputs. We consider an arbitrary system of differential-algebraic equations (DAE) in the form:

$$\dot{x}_j(t) = f_j(t, \theta, x(t)) \qquad j = 1, ..., n_x, \;\; t \in [t_0, +\infty) \tag{15}$$

$$x_j(t_0) = g_j(\theta) \tag{16}$$

$$y_k(t) = h_k(t, \theta, x(t)) \qquad k = 1, ..., n_y, \;\; t \in [t_0, +\infty)\,, \tag{17}$$

where $f : \mathbb{R} \times \Theta \times \mathbb{R}^{n_x} \to \mathbb{R}^{n_x}$, $g : \mathbb{R}^{n_p} \to \mathbb{R}^{n_x}$ and $h : \mathbb{R} \times \Theta \times \mathbb{R}^{n_x} \to \mathbb{R}^{n_y}$ are assumed to be a well defined measurable functions. Precise conditions guaranteeing solvability of the system (15)-(17) are beyond the scope of this paper, see for instance[2]. For simplicity of consideration, we expect f, g, and h to be regular enough, in particular, differentiable with respect to θ_i, $i \in \{1, ..., n_p\}$, and x_j, $j \in \{1, ..., n_x\}$, such that there exists a solution operator:

$$S : \Theta \to \mathscr{R}^{n_y}, \text{ where } S[\theta] = h(\cdot, \theta, x_\theta) \tag{18}$$

and x_θ solves the initial value problem (15)-(16).

Suppose that components of y represent observable quantities and let $\{(t_l, \tilde{y}_l)\}_{l \in \{1,...,m\}}$ denote the set of observations gathered at the prescribed points of time $\{t_l\}_{l \in \{1,...,m\}}$. Notice that $\tilde{y}_l$ is a vector with n_y components. We write $\tilde{y}_{k,l}$ in order to

refer to the k'th component of l'th measurement. Furthermore, let $\{\varepsilon_l\}_{l\in\{1,\ldots,m\}}$ be the corresponding measurement errors, i.e., the true values of observables, y^*, can be written as

$$y^*_k(t_l) = \tilde{y}_{k,l} + \varepsilon_{k,l}, \qquad k = 1,\ldots,n_y,\ l = 1,\ldots,m. \tag{19}$$

We do not assume that all the measurement errors are independent random variables but require their normal distribution with zero mean value, i.e. $\varepsilon \sim N(0, C_\varepsilon)$, where $C_\varepsilon \in \mathbb{R}^{n_y m \times n_y m}$ is a symmetric and positive definite covariance matrix. In the case of independent measurements, C_ε is a diagonal matrix with error variances on the main diagonal.

The task is to get from an estimate of the parameters θ to the true parameter θ^* which creates the error-free output, i.e. $S[\theta^*] = y^*$. To be more precise, let us first introduce the so-called residual function $r : \Theta \times \mathbb{R}^{n_y m} \to \mathbb{R}^{n_r}$ acting on the space of parameters and measurements. The true parameter θ^* and the perfect measurement y^* satisfy

$$r(\theta^*, y^*) = r(\theta + \delta, \tilde{y} + \varepsilon) = 0\,, \tag{20}$$

where δ is the correction of the initial guess θ we are looking for. A common example of such residual is

$$r_{k,l}(\theta, y) = S_k[\theta](t_l) - y_{k,l},\ \ k \in \{1,\ldots,n_y\},\ l \in \{1,\ldots,m\}\,. \tag{21}$$

Note that r is in general nonlinear in θ. It is therefore justified to use an iterative scheme. Let θ be our approximation of model parameters and $\tilde{y}$ be the measurement at hand. We now try to find a corrections δ and ε such that

$$0 = r(\theta + \delta, \tilde{y} + \varepsilon) \approx r(\theta, \tilde{y}) + J_{r,\theta}(\theta, \tilde{y})\delta + J_{r,\tilde{y}}(\theta, \tilde{y})\varepsilon, \tag{22}$$

where $J_{r,\theta}$ and $J_{r,\tilde{y}}$ denote Jacobian matrices of r with respect to the first and second argument respectively. Each single parameter θ_i, $i \in \{1,\ldots,n_p\}$ can be estimated in another process or come from the previous iteration of our program. In the former case, we assume that $\delta_i \sim N(0, \sigma_i)$. In the latter, on the other hand, we have no information about the real value of parameters and do not know what is their relation to others. This behavior is modeled by the vanishing inverse covariance matrix. Without loss of generality, we can assume that $\theta = [\theta^e, \theta^u]$, where the superscripts ^{e} and ^{u} refer to the first and second group of parameters and the inverse of the corresponding covariance matrix reads

$$C_\delta^{-1} = \begin{bmatrix} C_{\delta^k} & 0 \\ 0 & 0 \end{bmatrix}. \tag{23}$$

Another observation is that r can principally have many zeros. In the literature, there are different ways to find reasonable estimates[3]. In this consideration, we focus our attention on the maximum likelihood approach[3,4]. This is a special example of extremum estimate, where the objective to be maximized is the joint probability density

$$\rho(\delta, \varepsilon) \sim exp\left[-\frac{1}{2}\left(\delta^T C_\delta^{-1}\delta + \varepsilon^T C_\varepsilon^{-1}\varepsilon\right)\right]. \tag{24}$$

In many applications, it is necessary to introduce constraints into our system. Our calibration package allows for the bound constraints $\underline{\theta} \le \theta \le \overline{\theta}$, which are handled with the penalty method, and arbitrary equality constraints in the implicit form $c(\theta + \delta) = 0$.

Taking the constraints into account, as well as the fact that the exponential function in (24) is strictly increasing, the overall problem to be solved at every iteration of our algorithm is

$$\begin{aligned} \min_{(\delta,\varepsilon)\in\mathbb{R}^p\times\mathbb{R}^{n_y m}} \quad & Q(\delta, \varepsilon) := \frac{1}{2}\begin{bmatrix}\delta \\ \varepsilon\end{bmatrix}^T \begin{bmatrix} C_\delta^{-1} & 0 \\ 0 & C_\varepsilon^{-1}\end{bmatrix}\begin{bmatrix}\delta \\ \varepsilon\end{bmatrix} + P(\theta + \delta) \\ \text{s.t.} \quad & r(\theta + \delta, \tilde{y} + \varepsilon) = 0 \\ & c(\theta + \delta) = 0 \end{aligned} \tag{25}$$

where P is an extra term penalizing violations of the bound constraints.

Solution strategy

In the outer loop of our algorithm, we use a version of the Newton solver. Therefore, it is very important to find a good initial guess for the model parameters. These are usually initialized with values being experienced in the real world, as they often represent physical quantities, or with statistical tools. Furthermore, in order to evaluate the residual function r in (21) we need a solution to the initial value problem (15)-(16). This system of ordinary differential equations is treated with the

fourth-order Runge-Kutta method[5]. Finally, problems of type (25) in every iteration of our program are solved with the method of Lagrange multipliers. Linearizing at the current approximation $(\theta, \tilde{y})$ and applying the first-order optimality conditions we get the following linear system

$$\begin{bmatrix} C_\delta^{-1} & 0 & J_{r,\theta}^T & J_{c,\theta}^T \\ 0 & C_\varepsilon^{-1} & J_{r,\tilde{y}}^T & 0 \\ J_{r,\theta} & J_{r,\tilde{y}} & 0 & 0 \\ J_{c,\theta} & 0 & 0 & 0 \end{bmatrix} \begin{bmatrix} \delta \\ \varepsilon \\ \lambda \\ \mu \end{bmatrix} = \begin{bmatrix} -\nabla P(\theta) \\ 0 \\ -r(\theta,\tilde{y}) \\ -c(\theta) \end{bmatrix} \tag{26}$$

with Lagrange multipliers $\lambda \in \mathbb{R}^{n_y m}$ and $\mu \in \mathbb{R}^{n_c}$. We solve it using sparse LU factorization knowing that better solvers are on the market, which are commercial.

Implementation and interface

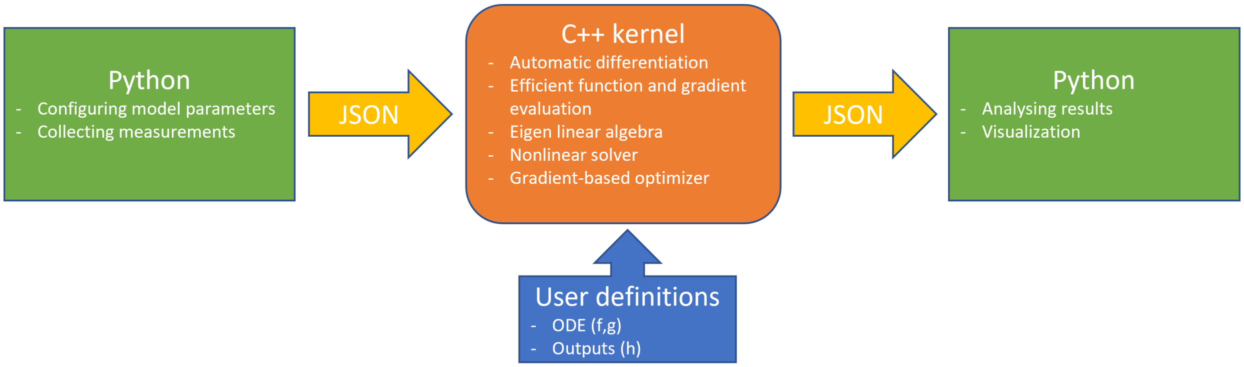

Figure 1. AD-Indent: interface

Our software AD-Ident is a two-term package, where the main computational kernel is implemented in C++ but the pre- and post-processing are made in Python, cf. Figure 1. Using such a strategy gives several important benefits. First of all, as the hard computational task is written in C++ and pre-compiled, it gives a better performance. The kernel is equipped with our own library for automatic differentiation (AD). Therefore, one can evaluate gradients of user-defined functions without additional effort and use efficient gradient-based methods in the solution process. The software uses sparse matrices, allowing the management of large-scale problems and efficient linear algebra packages distributed in the Eigen library[6]. It is worth mentioning that the package is fully generalized using the principles of objective programming. We can solve a variety of different problems from different fields of activity with the same code. All one has to do is to implement the corresponding functions f, g, and h in (15)-(17) defining the evolution of states, initial conditions, and outputs respectively. Model parameters as well as measurements are read automatically from the input JSON file and instantiated in the C++ program as AD variables. Only minimal C++ language knowledge is required to use the software.

The remaining part of the interface is implemented in Python giving high flexibility and a user-friendly control system. Users can utilize their own Python modules and a large variety of open-source modules to prepare the mathematical model of the problem at hand, define constraints, and finally, analyze and visualize results.

References

1. Teschl, G. *Ordinary Differential Equations and Dynamical Systems*. Graduate studies in mathematics (American Mathematical Society, 2012).

2. Kunkel, P. & Mehrmann, V. *Differential-algebraic Equations: Analysis and Numerical Solution*. EMS textbooks in mathematics (European Mathematical Society, 2006).

3. Hayashi, F. *Econometrics / Fumio Hayashi* (Princeton University Press, Princeton, N.J., 2000).

4. Miura, K. An introduction to maximum likelihood estimation and information geometry. *Interdiscip. Inf. Sci. (IIS)* **17**, DOI: 10.4036/iis.2011.155 (2011).

5. Butcher, J. *Runge–Kutta Methods*, chap. 3, 143–331 (John Wiley & Sons, Ltd, 2016). https://onlinelibrary.wiley.com/doi/pdf/10.1002/9781119121534.ch3.

6. Guennebaud, G., Jacob, B. *et al.* Eigen v3. http://eigen.tuxfamily.org (2010).
